# A novel oncogenic role for urokinase receptor in leukemia cells: molecular sponge for oncosuppressor microRNAs

**DOI:** 10.18632/oncotarget.25597

**Published:** 2018-06-12

**Authors:** Anna Li Santi, Anna Gorrasi, Mariaevelina Alfieri, Nunzia Montuori, Pia Ragno

**Affiliations:** ^1^ Department of Chemistry and Biology, University of Salerno, Salerno, Italy; ^2^ Department of Translational Medical Sciences, Federico II University, Naples, Italy

**Keywords:** uPAR, urokinase receptor, ceRNA, AML

## Abstract

Urokinase receptor (uPAR) expression is up-regulated and represents a negative prognostic marker in most cancers. We previously reported that uPAR and CXCR4 can be regulated by common microRNAs in leukemia cells. Transcripts containing response elements for shared microRNAs in their 3’UTR may regulate their availability.

We investigated uPAR 3’UTR capability to recruit microRNAs, thus regulating the expression of their targets. uPAR 3’UTR transfection in KG1 leukemia cells up-regulates the expression of endogenous uPAR. Transfection of uPAR 3’UTR, inserted downstream a reporter gene, increases uPAR expression and simultaneously down-regulates the reporter gene expression. Transfection of uPAR 3’UTR also increases CXCR4 expression; accordingly, uPAR silencing induces down-regulation of CXCR4 expression, through a mechanism involving Dicer, the endoribonuclease required for microRNA maturation.

Transfection of uPAR 3’UTR also increases the expression of pro-tumoral factors and modulates cell adhesion and migration, consistently with the capability of uPAR3’UTR-recruited microRNAs to target several and different transcripts and, thus, functions.

Finally, we found 3’UTR-containing variants of uPAR transcript in U937 leukemia cells, which show higher levels of uPAR expression as compared to KG1 cells, in which these variants are not detected.

These results suggest that uPAR mRNA may recruit oncosuppressor microRNAs, allowing the expression of their targets.

## INTRODUCTION

The urokinase (uPA) receptor (uPAR) focuses uPA proteolytic activity on the cell membrane, thus promoting localized plasminogen activation and degradation of extracellular matrix (ECM) [1]. uPAR is also an adhesion receptor for vitronectin (VN) [2–3]. uPA and VN, upon binding uPAR and activation of uPAR co-receptors, induce proteolysis-independent intracellular signalling, regulating cell adhesion, migration, survival and proliferation [1, 4–6]. uPAR expression can be regulated by various factors, indicating the potentially complex nature of uPAR gene regulation, consistent with a role for this molecule in several biological activities [7]. uPAR gene has a strong promoter region, which contains consensus sequences for various transcription factors [7]. Post-transcriptional regulation of uPAR expression via mRNA-binding proteins has been previously demonstrated [8–11].

Several types of non-coding RNAs have been identified in the last decade, and represent most of the human transcriptome [12]; non-coding transcripts include microRNAs (miRs). MiRs regulate gene expression by pairing with response elements generally located in the 3’UTR of target mRNAs, thus inhibiting transcript translation and, often, inducing their degradation [13]. MiRs play key roles in many biological processes and are aberrantly expressed in several malignancies. Indeed, some miRs can act as oncosuppressor genes, down-regulating the expression of specific oncogenes, whereas other miRs can act as oncogenes, down-regulating oncosuppressor genes. On these basis, miR levels have rapidly emerged as valuable markers for cancer diagnostics and promising targets in therapeutics; preclinical and clinical trials have been initiated for miRNA based therapeutics [14].

Emerging evidences of RNA-RNA crosstalk indicate new layers of gene expression regulation. It has been proposed in recent years that transcripts, which contain response elements for shared miRs, may regulate each other by titrating miR availability, thus acting as competing endogenous RNAs (ceRNAs). This hypothesis has been initially demonstrated for the non-coding PTENP1 *and* KRAS1P pseudogenes, whose transcripts can bind to and compete for the same pool of miRs bound by the transcripts of their ancestral genes PTEN and KRAS [15]. Since then, other ceRNAs have been validated, including other pseudogenes transcripts, some mRNAs, long non-coding (lnc) RNAs, circular (circ) RNAs [16]. RNAs with competing activity have been reported in various tumor types; in hematological malignancies, BRAFP1 pseudogene, several lncRNAs and c-myc mRNA have been reported as ceRNAs [17].

Overall, these findings suggest a powerful and unexpected biological activity for the different RNA types.

Recently, we identified oncosuppressor miRs able to target uPAR mRNA in acute myeloid leukemia (AML) cells [18]; thus, uPAR mRNA may participate to the RNA network regulating gene expression. In particular, since uPAR is up-regulated in most cancers, its transcript could act as a natural sponge for specific oncosuppressor miRs, allowing the translation of their oncogenic targets. Interestingly, variants of uPAR transcripts, containing the 3’UTR, have been identified in some tissues and cell lines, including peripheral blood mononuclear cells (PBMCs) and the AML THP-1 cell line [19].

We then explored the possible ceRNA activity of the 3’UTR of uPAR mRNA in AML cells and the functional implications of this activity.

## RESULTS

### uPAR 3’UTR up-regulates expression and function of endogenous uPAR

miRs are multitarget regulators of gene expression. We recently identified two miRs, miR-146a and miR-335, regulating uPAR expression in AML cell lines and in AML blasts. Indeed, these miRs directly target the 3’UTR of uPAR-mRNA and are highly expressed in KG1 acute myelogenous leukemia cells, which express low amounts of uPAR [18]. We hypothesized that transfection of uPAR-3’UTR could be able to recruit endogenous miR-146a and miR-335 and, possibly, other not yet identified miRs, competing with their targets, including endogenous uPAR mRNA, thus allowing their expression.

**Figure 1:**
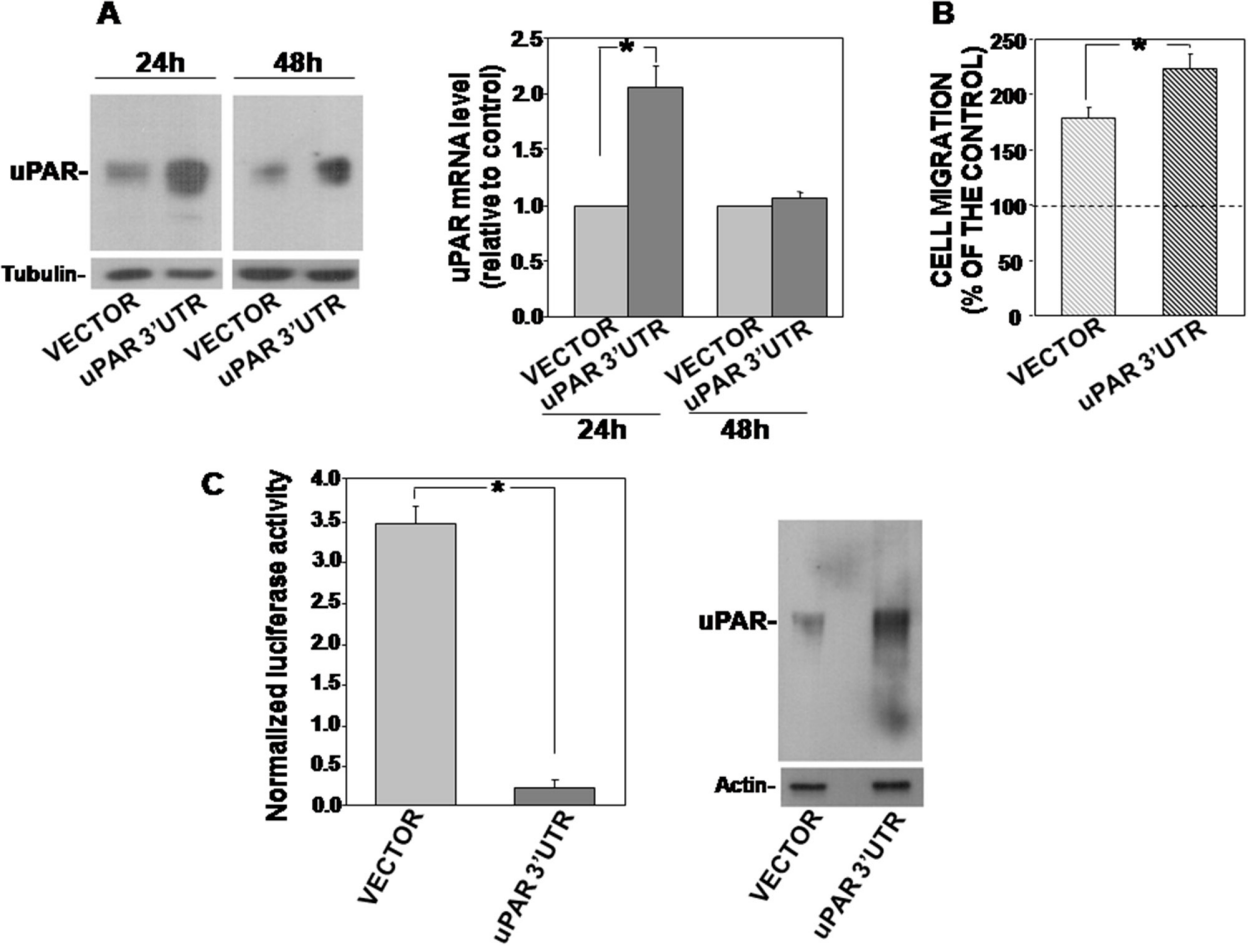
uPAR 3’UTR up-regulates endogenous uPAR expression and function and down-regulates the expression of the firefly-luciferase reporter gene **(A)** KG1 cells were transfected with uPAR 3’ UTR or the empty vector; after 24h and 48h cells were lysed and 50 μg of cells lysates were analyzed by Western blot with an uPAR specific antibody; filters were reprobed with mouse anti-tubulin antibody for loading control (left panel). Cells transfected with uPAR 3’UTR or the empty vector were also lysed in Qiazol and analyzed by qRT-PCR with primers specific for uPAR and GAPDH (for normalization) (right panel). Results are expressed as fold change of uPAR expression respect to cells transfected with the empty vector. Values are the mean ±SEM of three experiments performed in triplicate; (^*^) p≤0.05 as determined by the Student’s *t* test. **(B)** KG1 cells, 24h after transfection with uPAR 3’ UTR or the empty vector, were loaded in Boyden chambers and allowed to migrate toward 5 nM uPA aminoterminal fragment (ATF) for 2h. Migrated cells were fixed, stained with hematoxylin and counted. Results of migration assays are expressed as percentage of cells migrated towards the chemoattractant over the cells migrated without chemoattractant; 100% value represents cell migration in the absence of chemoattractant. The values are the mean±SEM of three experiments performed in triplicate. (^*^) p≤0.05, as determined by the Student’s *t* test. **(C)** KG1 cells were transiently co-transfected with uPAR 3’UTR cloned into the *firefly* luciferase-expressing pGL3 vector or the empty vector, and with the pRLSV40 vector, containing the *Renilla*-luciferase gene, as transfection efficiency control. Cells were lysed after 24h and assayed for the relative *Firefly* luciferase activity normalized to the internal control *Renilla*-luciferase (left) or analyzed by Western blot with an uPAR-specific antibody (right). Values are the mean ±SD of three experiments performed in triplicate. (^*^) p≤0.05 as determined by the Student’s *t* test.

We transiently transfected KG1 cells with the 3’UTR of uPAR or with the empty vector as control; transfected cells were harvested after 24h and 48h, lysed and analyzed by Western blot with uPAR specific antibodies. Western blot analysis showed that uPAR 3’ UTR induced an up-regulation of endogenous uPAR expression as compared to the empty vector both at 24h and 48h after transfection (Figure 1A, left). uPAR mRNA levels in transfected cells were also evaluated to verify that increased uPAR content at protein level was due to mRNA stabilization and not to delayed degradation of the protein. qRT-PCR analysis of uPAR mRNA levels in 3’UTR-transfected cells showed that uPAR increase at protein level corresponded to increased uPAR mRNA level at 24h from transfection (Figure 1A, right).

We then assessed whether uPAR 3’UTR transfection affects the levels of miRs targeting uPAR in this cell line [18], thus indirectly increasing uPAR expression. Indeed, uPAR 3’UTR transfection did not change the levels of examined miRs (not shown).

uPA, upon binding to uPAR, is able to induce migration of uPAR expressing cells. To examine the functionality of increased uPAR, chemotaxis assays were performed, showing that KG1 cells, transfected with uPAR 3’UTR, migrated more efficiently towards a specific uPAR ligand, the aminoterminal fragment of uPA (ATF), as compared to control cells, consistently with the increased expression of the receptor (Figure 1B).

All together, these results demonstrate that the 3’UTR of uPAR mRNA, devoid of any coding sequence, is able to induce the increase of uPAR expression and of uPAR-dependent cell migration.

### uPAR 3’UTR up-regulates uPAR expression by recruiting endogenous negative regulators of uPAR expression

We explored the mechanism regulating the increase of endogenous uPAR expression induced by uPAR 3’UTR transfection. To investigate whether uPAR 3’UTR is able to regulate the expression of a reporter gene, we used the uPAR 3’UTR-PGL3 construct in which the 3’UTR of uPAR is inserted immediately downstream the stop codon of the *firefly*-luciferase.

uPAR 3’UTR-PGL3 or the empty vector and the pRLSV40 vector, containing the *Renilla*-luciferase gene (for transfection efficiency normalization), were transiently co-transfected in KG1 cells; cells were then harvested and lysed after 24h. Cell lysates were assayed for *firefly*-luciferase activity or analyzed by Western blot for uPAR expression. Indeed, uPAR 3’UTR-PGL3 strongly down-regulated *firefly*-luciferase activity as compared to control cells transfected with the empty vector (Figure 1C, left), indicating that uPAR 3’UTR regulated negatively the expression of the reporter gene; at the same time, the uPAR 3’UTR increased the endogenous expression of uPAR (Figure 1C, right), as previously shown (Figure 1A). Thus, the observed increase of endogenous uPAR expression in uPAR 3’UTR-transfected cells was accompanied by the negative regulation of the reporter gene expression.

These results suggest that uPAR 3’UTR recruits endogenous factors able to down-regulate the expression of its upstream coding sequence, thus disengaging the endogenous targets of these cellular factors, including uPAR, whose expression results increased.

### uPAR 3’UTR regulates the expression of CXCR4

We hypothesized that the endogenous factors recruited by uPAR 3’UTR could be uPAR-targeting miRs. Indeed, we recently reported that three miRs which regulate uPAR expression, i.e. miR-146a, miR-335 and miR-622, all highly expressed in KG1 cells, also regulate the expression of the chemokine receptor CXCR4 [18]. Thus, we investigated whether uPAR 3’UTR regulates also the expression of CXCR4 beside uPAR expression.

**Figure 2:**
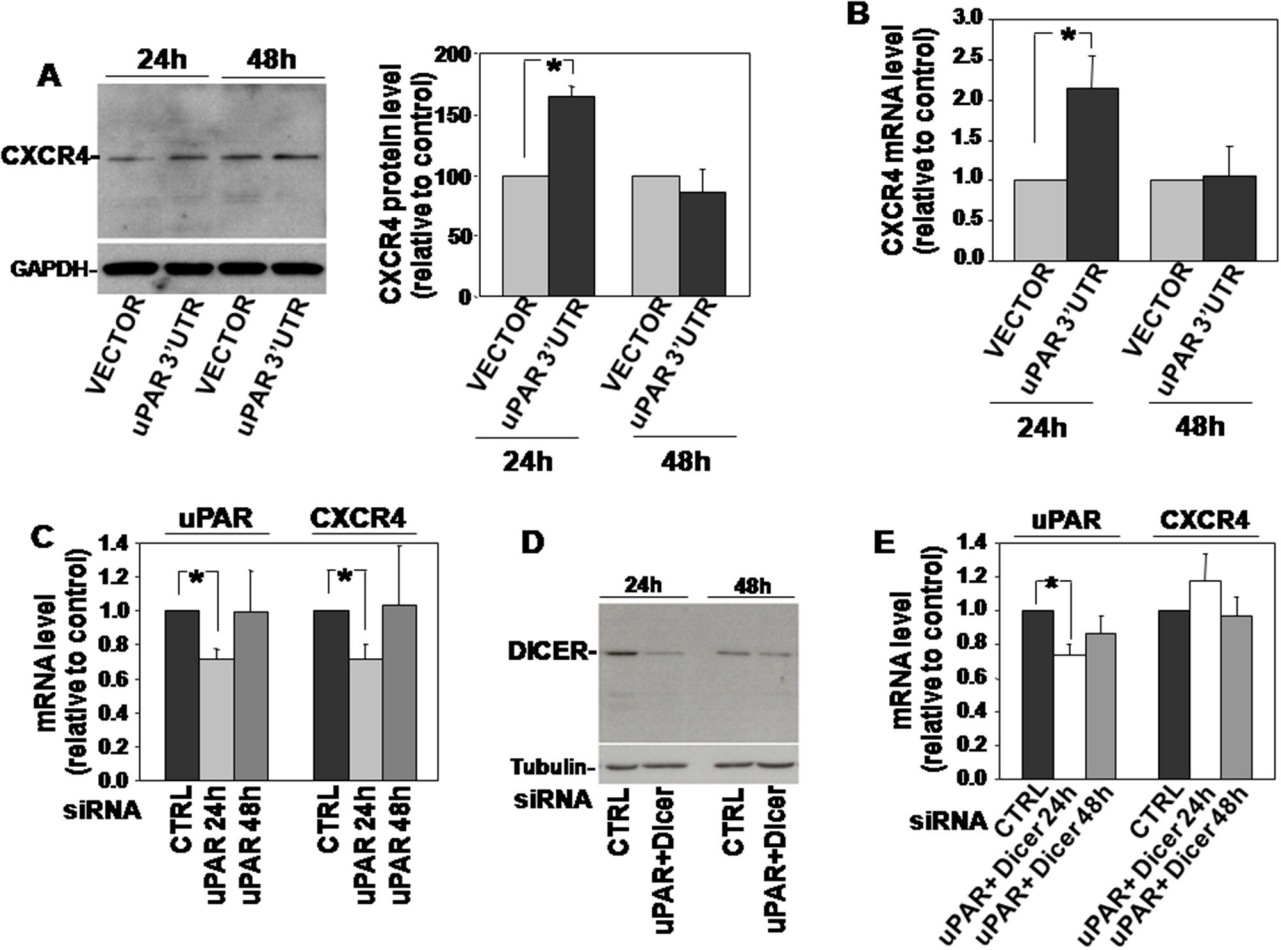
uPAR 3’UTR regulates the expression of CXCR4 **(A)** KG1 cells were transfected with uPAR 3’UTR or the empty vector; after 24h and 48h cells were lysed and 20μg of lysates were analyzed by Western blot with a CXCR4 specific antibody; filters were reprobed with a rabbit anti-GAPDH antibody for loading control (left panel). Detected bands were analyzed by densitometric scanning and the O.D. corresponding to CXCR4 bands were normalized to the O.D. of corresponding GAPDH bands (right panel: means ± S.E.M. of four separate experiments); (^*^) p≤0.05 as determined by the Student’s *t* test. **(B)** Part of cells transfected with uPAR 3’UTR or the empty vector were lysed in Qiazol and analyzed by qRT-PCR with primers specific for CXCR4 and GAPDH (for normalization). Results are expressed as fold change of CXCR4 expression respect to cells transfected with the empty vector. **(C)** KG1 cells were transfected with a uPAR targeting siRNA or with a control siRNA (CTRL); then, transfected cells were lysed in Quiazol at 24h and 48h and analyzed by qRT-PCR with uPAR or CXCR4 specific primers, and GAPDH primers for normalization. Results are expressed as fold change of uPAR or CXCR4 expression respect to cells transfected with the control siRNA. **(D)** KG1 cells were co-transfected with a uPAR- and a Dicer-specific siRNA or with a control siRNA; then, transfected cells were lysed in TRITON-X100 and 30μg of lysates were analyzed by Western blot with Dicer-specific antibodies. **(E)** KG1 cells were co-transfected with a uPAR- and a Dicer-specific siRNA or with a control siRNA; then, transfected cells were lysed in Quiazol at 24h and 48h from transfection and analyzed by qRT-PCR with uPAR or CXCR4 specific primers, and GAPDH primers for normalization. Results are expressed as fold change of uPAR or CXCR4 expression respect to cells transfected with the control siRNA.

KG1 cells were thus transiently transfected with uPAR 3’UTR and analyzed for CXCR4 expression. Western blot analysis and qRT-PCR analysis of transfected cells showed an increase in CXCR4 expression as compared to control cells 24h after transfection, both at protein and mRNA levels (Figure 2A and 2B, respectively); no significant effects were observed 48h after transfection.

We then adopted the opposite approach, that is silencing of uPAR expression in the same cell line and analysis of CXCR4 expression. In this case, the reduced levels of endogenous uPAR mRNA (which includes its 3’UTR) should make available higher levels of miRs targeting CXCR4, leading to a lower CXCR4 expression. Indeed, KG1 cells were transiently transfected with a uPAR-specific siRNA, which significantly reduced uPAR mRNA level after 24h, as demonstrated by qRT-PCR analysis; qRT-PCR analysis of CXCR4 expression on same transfected KG1 cells showed a parallel significant decrease of the CXCR4 transcript (Figure 2C). qRT-PCR analysis performed 48h after siRNA transfection showed that uPAR-mRNA level raised to basal level and, in parallel, also CXCR4 mRNA did the same (Figure 2C).

To explore the hypothesized involvement of miRs in this effect, we co-trasfected KG1 cells with the same uPAR siRNA and with a siRNA targeting the endoribonuclease Dicer, which is required for miRs biogenesis [13]. Dicer expression was strongly reduced 24h after transfection, as assessed by Western blot analysis with a Dicer specific antibody (Figure 2D). qRT-PCR analysis of uPAR-mRNA level confirmed the significant decrease of uPAR mRNA level 24h after uPAR-siRNA co-transfection, as expected; as hypothesized, uPAR silencing did not exert any effect on CXCR4-mRNA level in the absence of Dicer, and, thus, of mature miRs (Figure 2E). In both cases, no effects were observed 48h after transfection (Figure 2E).

Thus, in KG1 cells, transfection of uPAR 3’UTR increases the expression also of CXCR4 beside that of uPAR; accordingly, the decrease of the endogenous uPAR mRNA, which includes its regulatory 3’UTR, down-regulates the level of CXCR4 mRNA. uPAR 3’UTR-dependent negative regulation of CXCR4 mRNA occurs through a Dicer-dependent mechanism, suggesting miR involvement.

All together, these results support the hypothesis that transfected uPAR 3’UTR up-regulates uPAR and CXCR4 expression by recruiting endogenous miRs targeting both receptors, thus allowing the translation of their transcripts.

### uPAR 3’UTR modulates expression of various pro-tumoral proteins

Previous experiments focused on the effects of uPAR 3’UTR on the expression of uPAR itself and of CXCR4, which is known to be co-regulated with uPAR by common miRs in leukemia cells [18]. However, each miR regulates the expression of various targets; thus, miR recruitment by uPAR 3’UTR may affect the expression of other targets. Since uPAR expression is related to tumor progression, to identify some of these potential targets, we assayed lysates of uPAR 3’UTR transfected KG1 cells with the HumanXL Oncology Antibody Array (R&D); lysates and TCA-precipitated supernatants of transfected cells were also assayed with the Human Cytokine Antibody Array.

**Figure 3:**
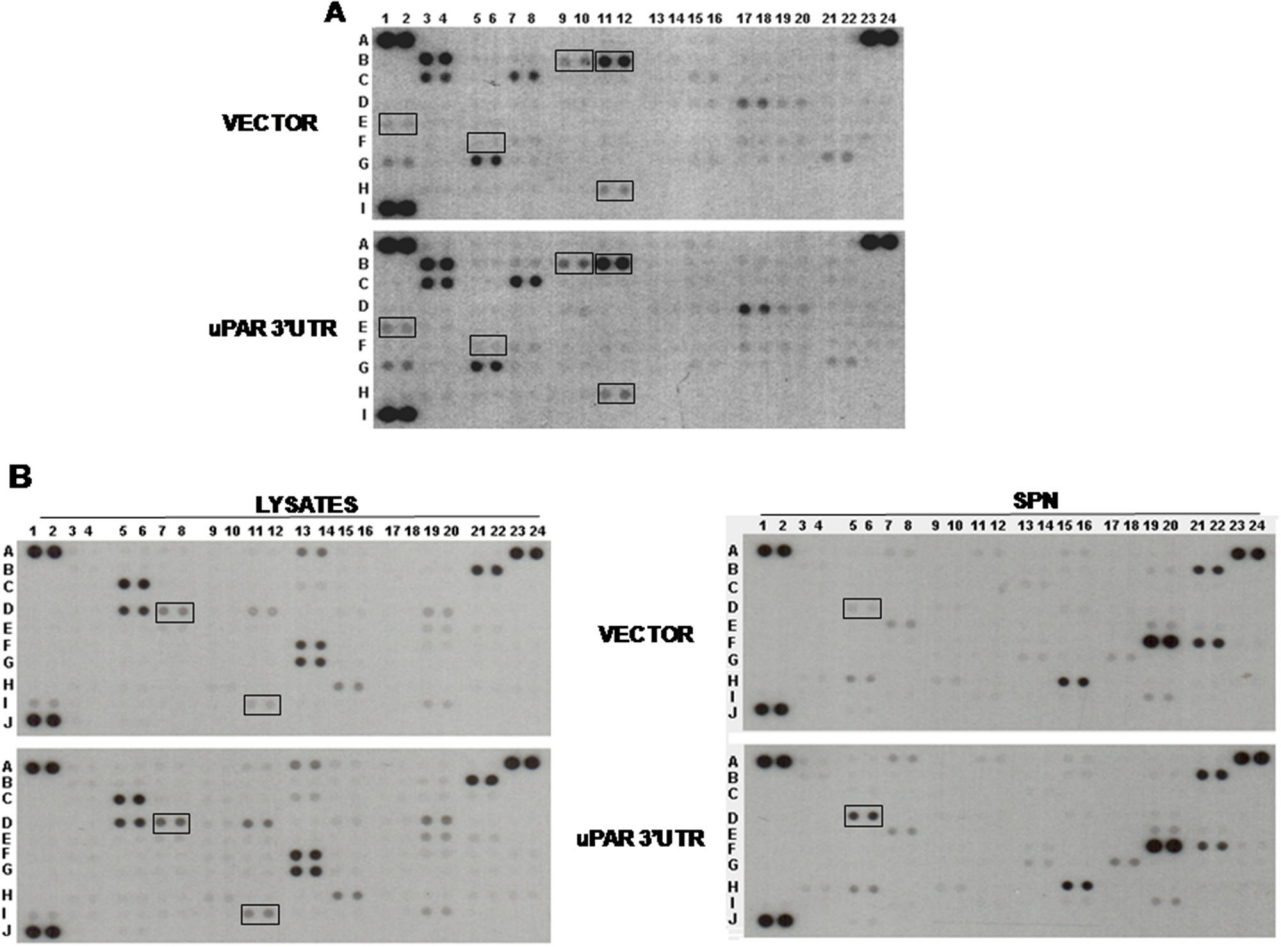
uPAR 3’UTR modulates expression of pro-tumoral proteins KG1 cells were transiently transfected with uPAR 3’UTR or the empty vector; at 24h from transfection, supernatants were harvested and cells were lysed in TRITON-X100. 200 μg of cell lysates were analyzed by the Human XL Oncology Antibody Array **(A)**. 200 μg of cell lysates and 2.5 ml of corresponding supernatants (SPN) were also analyzed by the Human Cytokine Antibody Array **(B)**. Boxes mark proteins selected for validation.

**Figure 4:**
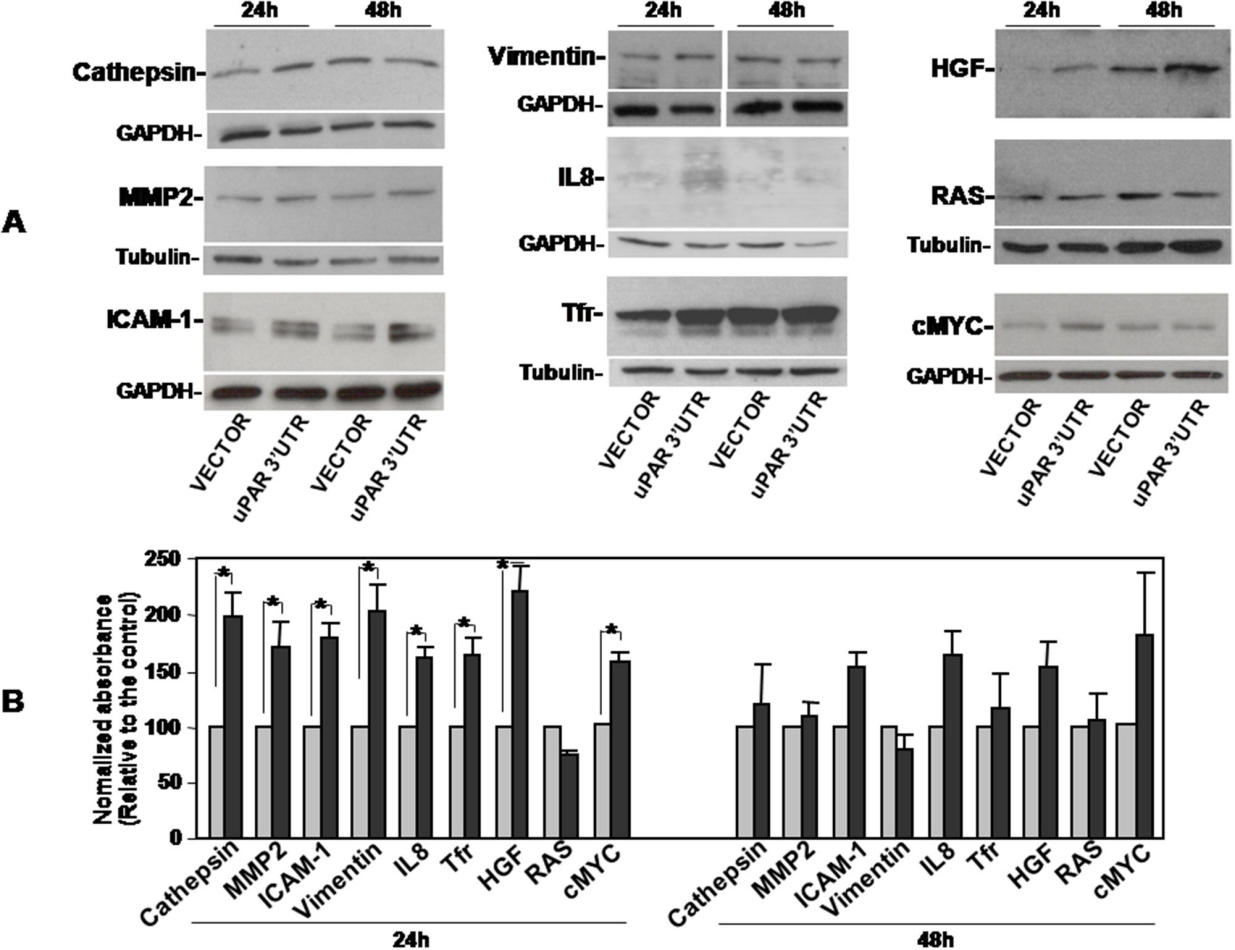
Validation of uPAR 3’UTR-induced up-regulation of pro-tumoral proteins expression KG1 cells were transiently transfected with the uPAR 3’UTR or the empty vector; after 24h and 48h cells were lysed. 20μg of cell lysates were analyzed by Western blot with antibodies against Cathepsins, MMP2 or ICAM-1; 10 μg, 40 μg and 5 μg of cell lysates were analyzed by Western blot with antibodies against IL-8, Vimentin and TfR1, respectively. Filters were reprobed with rabbit anti-GAPDH or mouse anti-tubulin antibodies as loading controls **(A)**. Detected bands were analyzed by densitometric scanning and the O.D. corresponding to specific bands were normalized to the O.D. of corresponding GAPDH or tubulin bands (means ± S.E.M. of three separate experiments; (^*^) p≤0.05 as determined by the Student’s *t* test) **(B)**.

Both arrays showed that uPAR 3’UTR induced variations in the expression of various factors, albeit to different extents. In Oncology Array, we validated cathepsins (B 9/10 and B11/12), IL-8 (E1/2), MMP2 (F5/6), Vimentin (H11/12) variations in cell lysates (Figure 3A); in Cytokine array, we validated variations of ICAM1 (D7/8) and TfR1 (I 11/12) in cell lysates (Figure 3B, left) and HGF variations in cell supernatants (D5/6) (Figure 3B, right). We confirmed the increase of these targets by Western blot analysis of lysates or supernatants of KG1 cells transiently transfected for 24h or 48h with uPAR 3’UTR or the empty vector (Figure 4). The significant increase occurred 24h after transfection and is more pronounced for IL-8, TfR1 and HGF.

We also analyzed cell lysates for the expression of well characterized oncogenes such Myc and RAS, finding no variations in RAS levels and, by contrast, up-regulation of Myc expression in uPAR 3’UTR transfected cells as compared to control cells (Figure 4).

To investigate whether observed increase in the expression of analyzed pro-tumoral factors may be due to the increase of uPAR expression rather than to the hypothesized mechanism, we analysed the expression of same factors in KG1 cells transfected with the uPAR cDNA lacking the 3’ UTR. Western blot analysis with specific antibodies showed that uPAR overexpression did not influence the levels of previously examined targets, further supporting a ceRNA activity of uPAR 3’UTR (not shown).

### uPAR 3’UTR influences cell adhesion and migration

MiRs regulate most biological processes, including cell adhesion, migration, proliferation [14].

We explored the possibility that uPAR 3’UTR, modulating the availability of specific miRs, influences these cell activities. To this end, KG1 cells were stably transfected with uPAR 3’UTR cloned in the PcDNA3.1(+) vector or with the empty vector as control.

**Figure 5:**
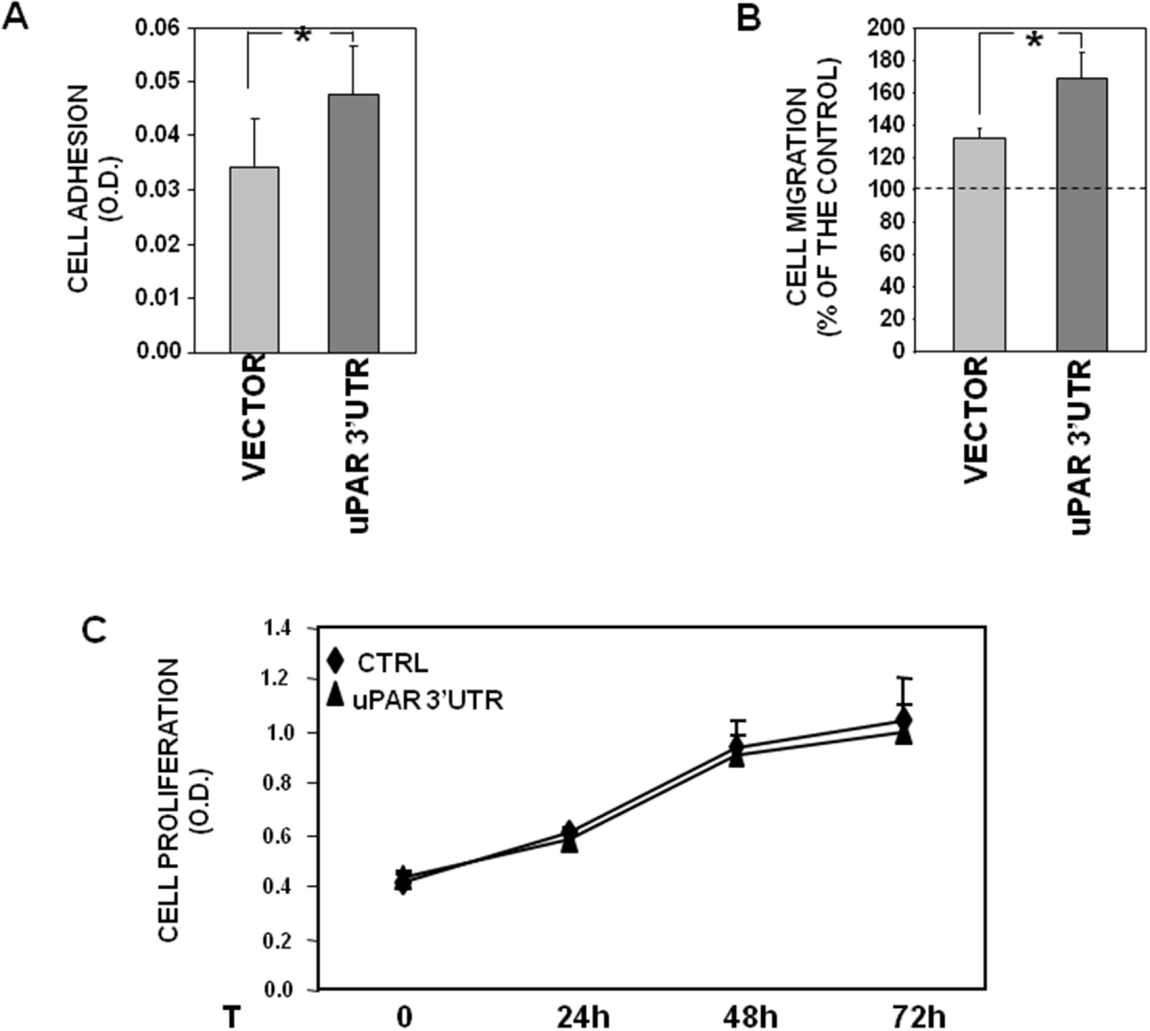
uPAR 3’UTR influences cell adhesion and migration **(A)** 1.5x10^5^ KG1 cells, stably transfected with uPAR 3’ UTR or the empty vector, were plated in wells pre-coated with 10 μg/ml of fibronectin (FN) or 1% BSA in PBS as a negative control, and incubated for 2h at 37°C, 5% CO_2_. Attached cells were fixed with 3% PFA and stained with crystal violet; stain was eluted and its absorbance at 540 nm was measured with a spectrophotometer. Values corresponding to cells plated on FN were subtracted of values corresponding to cells plated on BSA. The values are the mean ± SEM of six experiments performed in triplicate. (^*^) p≤0.05, as determined by the Student’s *t* test. **(B)** 2x10^5^ KG1 cells, stably transfected with uPAR 3’ UTR or the empty vector, were loaded in Boyden chamber and allowed to migrate towards 10% serum (FBS). Migrated cells were fixed, stained with haematoxylin and counted. Results of migration assays are expressed as percentage of cells migrated towards chemoattractants over the cells migrated without chemoattractants; 100% value represents cell migration in the absence of chemoattractants. The values are the mean ± SEM of five experiments performed in triplicate. (^*^) P ≤ 0.05, as determined by the Student’s *t*-test. **(C)** KG1 cells, stably transfected with uPAR 3’ UTR or the empty vector, were serum-starved for 16h and cultured with 5% FBS in IMDM; at 0, 24, 48 or 72h, 20μl/well of CellTiter 96 AQueous One Solution Reagent was added to each well and incubated for 4hrs at 37°C, 5% CO_2_. The absorbance was determined by an ELISA reader (Bio-Rad) at a wavelength of 490 nm. The values are the mean ± SEM of three experiments performed in quadruplicate.

First, capability of transfected cells to adhere to fibronectin (FN), a component largely present in stromas, was assayed, showing that uPAR 3’ UTR significantly increases KG1 cell adhesion as compared to control (Figure 5A).

Transfection of uPAR 3’UTR significantly increased also cell migration toward serum, as shown by cell migration assays in Boyden chambers (Figure 5B).

By contrast, no effects of uPAR 3’ UTR were observed on cell proliferation, since both 3’UTR- and vector-transfected cells showed the same proliferation rate up to three days (Figure 5C).

These results may suggest that the uPAR 3’UTR recruits mainly miRs involved in cell adhesion and migration rather than miRs involved in cell proliferation.

### Expression of variants of uPAR transcripts in leukemia cells

Finally, to further explore the mechanism underlying uPAR 3’UTR effects, we looked for uPAR transcript variants carrying this regulatory region, independently on their translation in protein.

**Figure 6:**
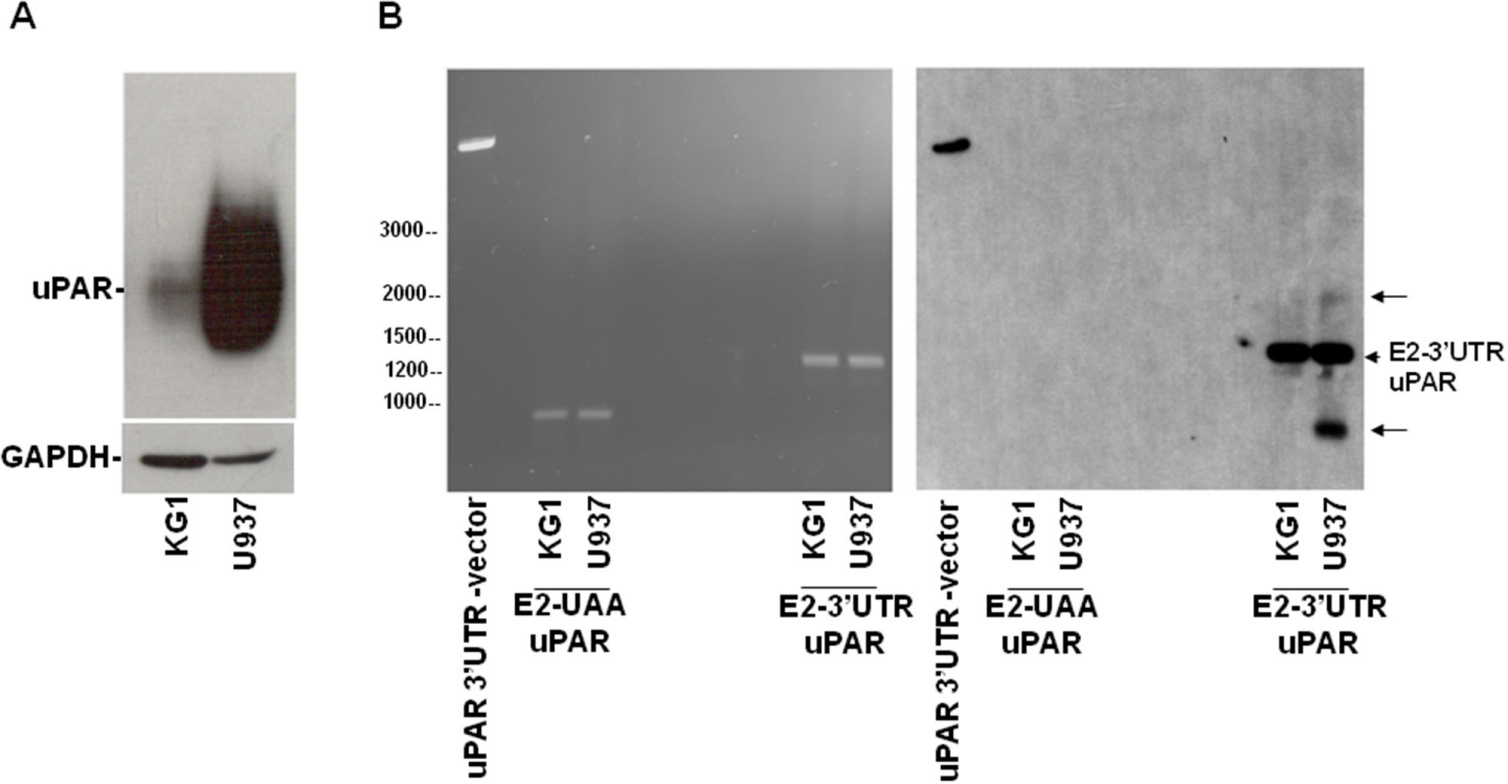
Expression of variants of uPAR transcripts in leukemia cells **(A)** KG1 or U937 cells were lysed and analyzed by Western blot with an anti-uPAR antibody; the filter was reprobed with an anti-GAPDH antibody for loading control. **(B)** KG1 and U937 cells were lysed in Quiazol Reagent and total RNA reversely transcribed; then, 2 μl of reversely transcribed DNA were used for PCR amplification of the region encompassing uPAR from Exon2 to the stop codon UAA (E2-UAA) or of the region encompassing uPAR from Exon2 to the whole 3’UTR (E2-3’UTR). 7 μg of PCR products and the linearized uPAR 3’UTR-PcDNA3.1, as positive control, were analyzed by electrophoresis in 1.2% agarose gel containing ethidium bromide, photographed under ultraviolet illumination (B, left panel), blotted to a Nylon membrane and hybridized with biotinylated 3’UTR RNA probe (B, right panel).

We focused on KG1 and U937 cells, expressing low and high levels of uPAR, respectively, as previously shown [18] and confirmed in Figure 6A; accordingly, KG1 and U937 cells express, respectively, high and low levels of uPAR-targeting miRs [18].

Total RNAs from both cell lines were reversely transcribed and used as templates for two different PCRs, one performed with primers designed to amplify uPAR from Exon 2 to the stop-codon (PCR products: E2-UAA uPAR), the other performed with primers designed to amplify uPAR from Exon 2 to the whole 3’UTR (PCR products: E2-3’UTR uPAR). Same amount (7μg) of PCRs products were electrophoresed (Figure 6B, left) and analyzed by Southern blot, probing the filter with labeled 3’UTR (Figure 6B, right). In the lanes containing the E2-3’UTR PCR products, a band corresponding to uPAR from Exon2 to the whole 3’UTR was detected in both cell lines; the corresponding band lacking the 3’UTR was not detected in the E2-UAA PCR products, as expected (Figure 6B, right). Interestingly, U937 cells showed two additional bands containing the 3’UTR, undetectable in KG1 cells, which may represent uPAR variants carrying the 3’UTR. Also these additional bands were not detected in the control PCR products lacking the 3’UTR, as expected (Figure 6B, right).

Thus, U937 cells seem to express uPAR transcript variants containing the uPAR 3’UTR, which may serve as decoy mRNAs for uPAR targeting miRs, promoting expression of uPAR and other targets.

## DISCUSSION

uPAR is potentially involved in most crucial events underlying tumorigenesis and tumor progression [20–21]. In fact, this cell surface protein can regulate cell adhesion and migration, since it is a vitronectin receptor, associates to integrins regulating their activity, concentrates the proteolytic activity of uPA, promoting the focalized ECM degradation required for cell migration through tissues [1, 20]. uPAR is also involved in cell proliferation and survival [22]. Accordingly, uPAR up-regulation represents a negative prognostic factor in various tumor types, including hematological malignancies as acute myeloid leukemia and multiple myeloma [23]. A comparative analysis between peripheral blood and bone marrow (BM) AML blasts at diagnosis and relapse revealed that uPAR expression was significantly higher in circulating blast cells and at relapse, suggesting that uPAR expression positively correlates with invasive manifestations of AML [23]. Further, uPAR is involved in hematopoietic stem cell mobilization and in their cross-talk with the bone marrow microenvironment [24–25].

Several efforts have been done to find strategies able to neutralize the multiple activities of this receptor [26–27]. However, here we aimed to investigate whether uPAR may be involved in pro-tumoral activities at a completely different level, that is at transcript level. Post-transcriptional regulation of uPAR expression has been previously reported; in fact, uPAR mRNA can bind proteic factors able to regulate its stability, as it occurs for other components of the plasminogen activator system [9–11]. We recently reported that uPAR expression can be regulated by three miRs, in particular by miR-146a and miR-335, endowed with oncosuppressor activity, expressed in leukemia cells and in AML blasts [18]. In last years, several evidence strongly supported the hypothesis that non-coding RNAs may form a highly complex network regulating gene expression [12], even with some criticisms and controversies [28]. All together, these emerging aspects about RNA types and functions prompted us to investigate whether uPAR may play new and unexpected role in cancer, in particular whether uPAR mRNA, which is target of oncosuppressor miRs, can act as a molecular sponge for them, thus promoting the expression of pro-tumoral genes.

We focused on the KG1 AML cell line as experimental system, since we previously demonstrated that, in these cells, the 3’UTR of uPAR mRNA is targeted by two highly expressed oncosuppressor miRs [18, 29–34].

We started to hypothesize that if transfected uPAR 3’UTR recruits uPAR-targeting miRs, endogenous uPAR mRNA should be saved for translation, leading to increased uPAR expression. In fact, we show that overexpression of uPAR 3’UTR fused to a reporter gene, increases the expression of endogenous uPAR, down-regulating the expression of the reporter gene. Then, we found that overexpression of uPAR 3’UTR also up-regulates the expression of a uPAR co-regulated gene, CXCR4 [18] and, accordingly, the silencing of uPAR mRNA, containing the endogenous regulatory sequence, down-regulates CXCR4 expression. These uPAR 3’UTR effects are abrogated by silencing Dicer, the enzyme required for miR biogenesis, suggesting miRs as the regulatory factors recruited by uPAR 3’UTR. Since miRs are multitarget molecules and uPAR 3’UTR may bind also not yet identified miRs, regulating the expression of their unknown targets, we extended our investigation to the regulation of pro-tumoral factors, using microarray kits, showing and validating a uPAR 3’UTR-dependent increase in the expression of proteases, as Cathepsins and MMP2, an iron metabolism receptor as the transferrin receptor (TfR1), the cytoskeleton component vimentin, the intercellular adhesion molecule-1 (ICAM-1), the pro-inflammatory chemokine Interleukin-8 (IL-8), and, finally, the oncogene Hepathocyte growth factor (HGF). Thus, uPAR 3’ UTR overexpression influences the expression of all these factors which are up-regulated and/or play a role in various tumors including AML [35–43]. Interestingly, uPAR 3’UTR also promotes cell adhesion and migration probably by regulating the expression of factors related to these biological processes.

Finally, we looked for uPAR transcript variants able to exert the hypothesized decoy activity of uPAR 3’ UTR, identifying two possible variants carrying the 3’UTR, thus capable to recruit uPAR-targeting miRs in U937 cells. These transcript variants are detected only in U937 cells, which express high uPAR levels as compared to KG1 cells, in which the corresponding bands are undetectable.

Indeed, uPAR transcript variants have been previously reported in various cell types, including PMN, PBMC, THP1 leukemia cells [19, 44]; further, uPAR variant del4/5 mRNA (lacking exons 4 and 5) has been proposed as a novel prognostic marker in breast cancer [45–46]. However, much attention has been paid to their translation into protein, whereas it is possible that these uPAR mRNA variants have their own roles, crucial in the regulation of gene expression.

All together these results suggest that uPAR mRNA can act as a ceRNA, participating to the RNA network regulating gene expression in leukemia cells, promoting pro-tumoral activities independently on its translation in protein. These observation also imply that, in therapeutical approaches, it is crucial to distinguish uPAR mRNA activities from uPAR protein activities, because targeting the protein may not block uPAR mRNA activities, leading to inefficient strategies.

## MATERIALS AND METHODS

### Cell culture

KG1 acute myelogenous leukemia cell line was cultured in IMDM supplemented with 20% heat-inactivated fetal bovine serum (FBS). U937 promonocytic leukemia cell line was cultured in RPMI 1640 supplemented with 10% heat-inactivated FBS.

### Transfections

The 319-bp fragment encompassing uPAR 3’UTR (http://genome.ucsc.edu/) was inserted in the XbaI site of the pGL3 vector, downstream the stop codon of *firefly*-luciferase reporter gene, as previously described [18]. uPAR 3’UTR was also cloned in XbaI site of the PcDNA3.1(+) vector for stable transfections. The constructs were checked by sequence analysis.

KG1 cells were transfected by electroporation using Amaxa™ Nucleofector™ Technology, according to the protocol specifically indicated by the manufacturer (Lonza). 2×10^6^ cells were transfected in 100μl of HBSS medium with 2μg of DNA or 100 nM siRNA uPAR or 200 nM siRNA Dicer (Santa Cruz), then diluted to 1.6ml and incubated for the indicated times.

In transfections for luciferase assays, 70 ng of pRLSV40 plasmid (Promega), containing the *Renilla*-luciferase, were co-trasfected for normalization of transfection efficiency.

### Luciferase assay

Cells were co-transfected with the pGL3-3’UTR/uPAR construct or the empty vector and pRLSV40 for normalization. After 24h, transfected cells were lysed and the luciferase activity was measured with a luminometer using the dual-luciferase reporter assay system (Promega) [18].

### Western blot analysis

Cells were lysed in 1% Triton X-100 and the protein content measured by a colorimetric assay (BioRad); indicated amounts of cell lysates were electrophoresed in SDS-PAGE, transferred onto a PVDF filter (Millipore), blocked with 5% milk and probed with primary antibodies. Washed filters were incubated with horseradish peroxidase-conjugated secondary antibodies (Bio-Rad) and bands detected by ECL (Amersham).

## RT-PCR

Cells were lysed in Qiazol (Life Technologies) and total RNA was isolated according to the supplier’s protocol. Total RNA (5μg) was reversely transcribed with random hexamer primers and 200 U of SSIII reverse transcriptase (Invitrogen). 2μl of reversely transcribed DNA were amplified for 30 cycles with 2.5 units of Taq polymerase (Invitrogen) using the forward primer 5’ CGGTGCATGCAGTGTAAGAC-3’ and the reverse primer 5’-TTAGGTCCAGAGGAGAGTGC-3’ to amplify uPAR from exon2 to the stop codon UAA (PCR products: E2-UAA), or using the same forward primer 5’ CGGTGCATGCAGTGTAAGAC-3’ and the reverse primer CCACTGGTACAAAATCTTTATG-3’ to amplify uPAR from exon2 to the whole 3’UTR (PCR products: E2-3’UTR).

For quantitative RT-PCR (qRT-PCR), 1 μg of total RNA was reverse-transcribed and 1 μl of a 1:10 dilution was analyzed by qRT-PCR with a BioRad IQ5 system, using IQTMSYBR Green Supermix for qPCR kit, according to manufacturer’s instructions. mRNAs levels were normalized to the glyceraldehyde-3-phosphate dehydrogenase (GAPDH) mRNA levels. Primers, designed using Primer3 software and used at 0.25 μM, were previously described [18]. The relative levels of expression were calculated with the formula 2^–ΔΔ^Ct.

### Southern blot

7μg of PCR products (see RT-PCR paragraph) were electrophoresed in 1.2% agarose gel containing ethidium bromide and photographed under ultraviolet illumination; then, the gel was incubated in 0.5 M NaOH, 1.5 M NaCl for DNA denaturation, neutralized and blotted to a Nylon membrane (Roche). The 3’UTR biotinylated RNA probe was prepared from 3’UTR-PcDNA3.1(+) with MAXIscript kit using T7 polymerase and BIO16UTP, according to the manufacturer’s instruction (Thermo Scientific). The membrane was incubated for 16h at 68°C with labeled 3’UTR RNA probe (10 ng/ml), in hybridization buffer (50% formamide, 6X SSC, 5X Denhardt’s solution, 0.5% SDS, 100μg/ml denatured salmon sperm DNA). Membrane was washed twice in 2X SSC, 0.5% SDS for 15 min at room temperature and twice in 0.1X SSC, 0.5% SDS for 60min at 60°C. Signal was detected by using the kit “Chemiluminescent nucleic detection module” (Thermo Scientific).

### Cell migration assay

Migration was performed in Boyden chambers, using uncoated PVPF polycarbonate filters (5μm pore size) (Whatman). 2×10^5^ cells were loaded in the upper chamber in serum-free medium; 5 nM ATF (American Diagnostica) or 10% FBS were added in the lower chamber as chemoattractants. Cells were allowed to migrate for 2h at 37°C, 5% CO2. Then, the cells on the lower surface of the filter were fixed in 70, 90, 100% ethanol, stained with Mayer’s hematoxylin, and counted at 20x magnification (10 random fields/filter).

### Cell adhesion assay

Flat-bottom 96-well microtiter plates were coated with 10μg/ml of fibronectin (Roche) or 1% heat-denatured BSA-PBS as a negative control, and incubated 16h at 4°C. The plates were then blocked 1h at room temperature with 1% heat-denatured BSA-PBS. 1.5×10^5^ cells were plated in each coated well and incubated for 2h at 37°C. Then, wells were washed and attached cells were fixed with 3% paraformaldehyde (PFA) at 37°C in PBS and then with 20% methanol. Cells were finally stained with 0.5% crystal violet in 20% methanol. Stain was eluted by 0.1 M sodium citrate in 50% ethanol, pH 4.2, and the absorbance at 540 nm was measured with a spectrophotometer.

### Cell proliferation assay

Cells were serum-starved for 16h and then cultured with 5% FBS in IMDM; at 0, 24, 48 or 72h cell samples were harvested, diluted in 400μl and distributed in four wells of 96-well plates. Then, 20μl/well of CellTiter 96 AQueous One Solution Reagent (Promega) was added and incubated for 4h at 37°C, 5% CO2. The absorbance was determined by an ELISA reader (BioRad) at a wavelength of 490 nm.

### Statistical analysis

Differences between groups were evaluated by the Student’s t test using PRISM software (GraphPad, San Diego, CA). *P*≤ 0.05 was considered statistically significant.
